# Human esophageal myofibroblast secretion of bone morphogenetic proteins and GREMLIN1 and paracrine regulation of squamous epithelial growth

**DOI:** 10.1038/s41598-018-30799-7

**Published:** 2018-08-17

**Authors:** Chunying Zhang, Chao Niu, Kevin Yang, Anisa Shaker

**Affiliations:** Department of Internal Medicine, Division of Gastrointestinal and Liver Diseases, Keck School of Medicine of USC, Los Angeles, CA USA

## Abstract

We have previously shown myofibroblasts subjacent to the squamous epithelium in the normal human esophagus and an increase in esophagitis. Myofibroblast contribution to bone morphogenetic protein (BMP) signaling and to paracrine mediated epithelial-mesenchymal interactions in the human esophagus remains incompletely defined. We investigated BMP4 and BMP inhibitor GREM1 gene expression and protein levels in previously characterized human esophageal myofibroblast primary cultures and in a human esophageal myofibroblast cell line. We adapted human esophageal myofibroblast conditioned media into a 3D organotypic model to investigate the effect of myofibroblast secreted factors on squamous epithelial morphology, proliferation, differentiation and BMP signaling. Human esophageal myofibroblasts constitutively secrete GREM1 and increase *BMP4* expression and BMP4 secretion in response to epithelial Hedgehog ligand SHH. Detection of secreted BMP4 is decreased in the presence of GREM1. Myofibroblast conditioned media increases epithelial proliferation and expression of basal markers p63 and CK14 leading to an overall increase in epithelial thickness. Epithelial BMP signaling increases with myofibroblast conditioned media. These findings were partially reversed with GREM1 inhibition. Our results demonstrate that myofibroblasts are potential sources of GREM1 and of BMP4 in the human esophagus and that human esophageal myofibroblast-epithelial paracrine interactions contribute in part to the regulation of epithelial growth.

## Introduction

The bone morphogenetic protein (BMP) pathway is required for embryonic esophageal epithelial morphogenesis^1^ and activation of BMP signaling activity has been found in esophagitis^2^, Barrett’s esophagus (intestinal metaplasia of the squamous epithelium)^3^ and most recently eosinophilic esophagitis^4^. Recent studies using genetic mouse models have shown that hyper-activation of BMP signaling promotes squamous differentiation while inhibition promotes expansion of basal progenitor cells^4^. On the other hand, others have shown that an increase in BMP activity is associated with intestinal differentiation^2^. Overall, it appears that the regulation of esophageal epithelial basal layer proliferation and differentiation by BMP signaling may be context specific^5^. Secreted BMPs induce heterodimeric BMP receptor type 1 and 2 complexes on target cells, leading to phosphorylation of intracellular receptor SMAD 1/5/8 via BMPR1 activation^6^. P-SMAD 1/5/8 then translocates with SMAD4 to the nucleus to transcribe target genes including inhibitor of differentiation (ID) 1–2^7^. BMP4 binds with greatest affinity to the type 1 BMP receptors BMPR1A and BMPR1B^6^ and its activity is regulated in part by secreted extracellular antagonists including Gremlin (GREM) 1, Follistatin (FST), Noggin (NOG), and Chordin (CHRD)^8^. BMP4 is also an end-target of the Hedgehog (Hh) signaling pathway^9^. Although BMP signaling has been described in the mouse esophageal epithelium^1–4^, the mesenchymal cell contribution to this pathway has been incompletely investigated in the human esophagus.

We have previously identified human esophageal myofibroblasts (HEMFs) subjacent to the squamous epithelium of the human esophagus and an increase in this population of cells in esophageal biopsies with histological characteristics of reflux esophagitis^10^. We have also previously established and characterized primary cultures of human esophageal myofibroblasts (HEMFs) and an immortalized HEMF cell line generated from normal human esophagus^11^. HEMFs secrete a number of inflammatory cytokines in response to stimulation^10^ and support the growth of squamous epithelial cells in 3D organotypic culture, in part by adding structural support to the collagen matrix^11^. The location of these cells coupled with their secretory capacity suggested a potential role for paracrine interactions between HEMFs and the squamous epithelium. We were curious whether HEMFs were a mesenchymal source of BMP4 and GREM1, a BMP inhibitor that has not been extensively described in the human esophagus. We were also interested whether paracrine mechanisms between HEMFs and the epithelium were involved in supporting the growth of the stratified squamous epithelium.

## Results

### BMP4 and GREMLIN1 gene expression and protein levels in unstimulated HEMFs

**Figure 1:**
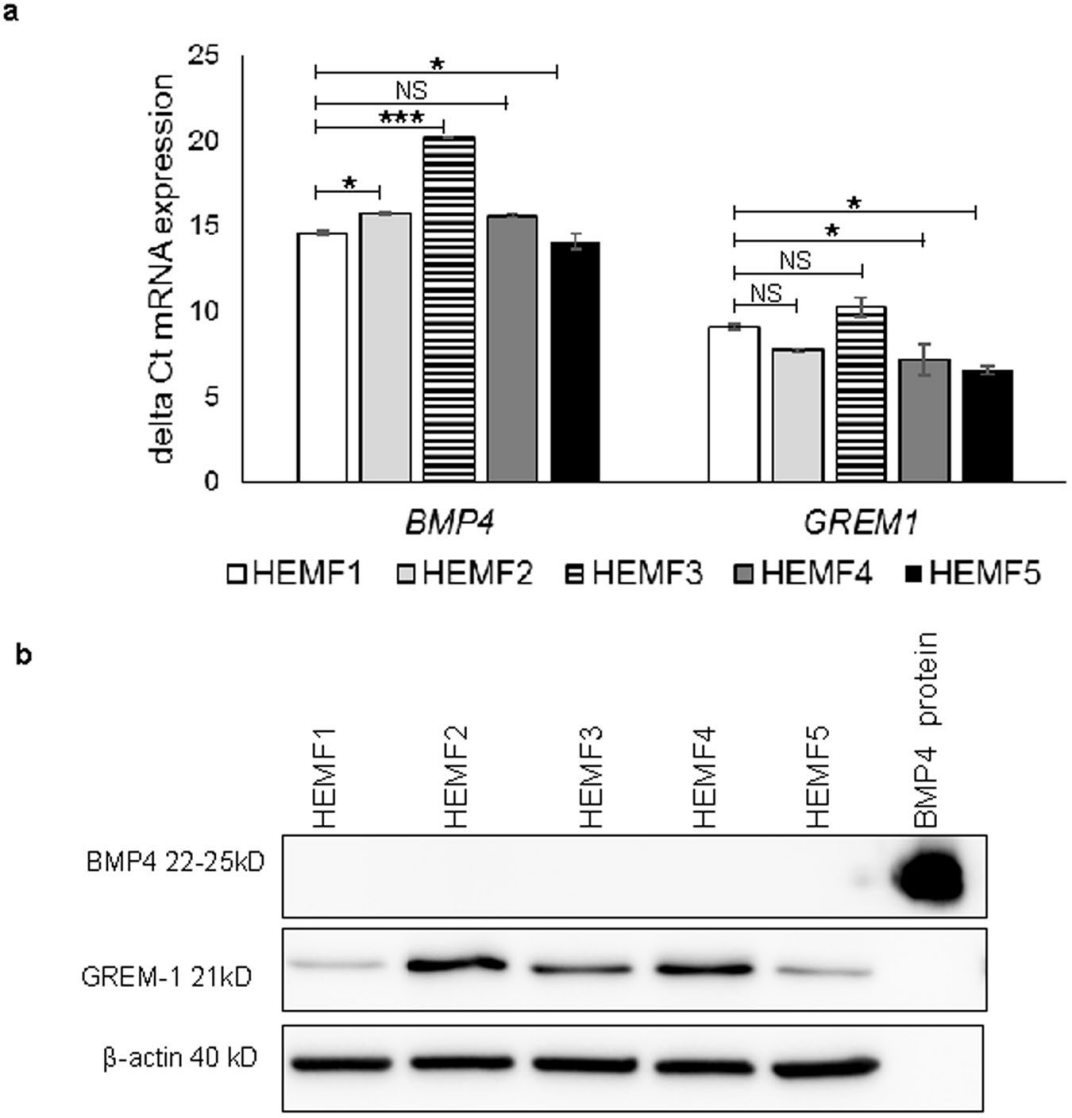
BMP4 and GREM1 gene expression and protein levels in unstimulated HEMFs. (**a**) *BMP4* and *GREM1* mRNA expression were measured by qRT-PCR performed on RNA isolated from primary cultures of HEMFs (HEMFs 1–4) established from normal human esophagi and from a GFP-hTERT transfected immortalized HEMF cell line (HEMF 5). Absolute and relative gene abundance are shown with delta Cts (∆Ct) wherein a higher delta Ct indicates lower gene expression. *P ≤ 0.02; **P ≤ 0.001; NS, non-significant vs. HEMF1. Values represent the mean ± SEM of three individual experiments. (**b**) Representative immunoblots for BMP4 and GREM1 performed on HEMFs 1–5 with β-actin as the loading control are shown. Detection of BMP4 recombinant protein as a positive control is also shown.

qRT-PCR showed baseline expression of *BMP4* and *GREM1* mRNA in primary HEMF cultures established from multiple normal human esophagi (HEMFs 1–4). As expected, absolute differences in mRNA expression of *BMP4* and *GREM1* were observed amongst HEMFs established from different individuals. However, *BMP4* and *GREM1* were readily detected in all samples. *BMP4* and *GREM1* mRNA expression were also confirmed in a previously described immortalized HEMF cell line (HEMF 5)^11^ (Fig. 1a). BMP4 and GREM1 protein expression were then evaluated in HEMF cell lysates. BMP4 protein could not be detected in untreated HEMFs. GREM1 protein expression was readily detected in all samples (Fig. 1b).

### BMP4 signaling in HEMFs

**Figure 2:**
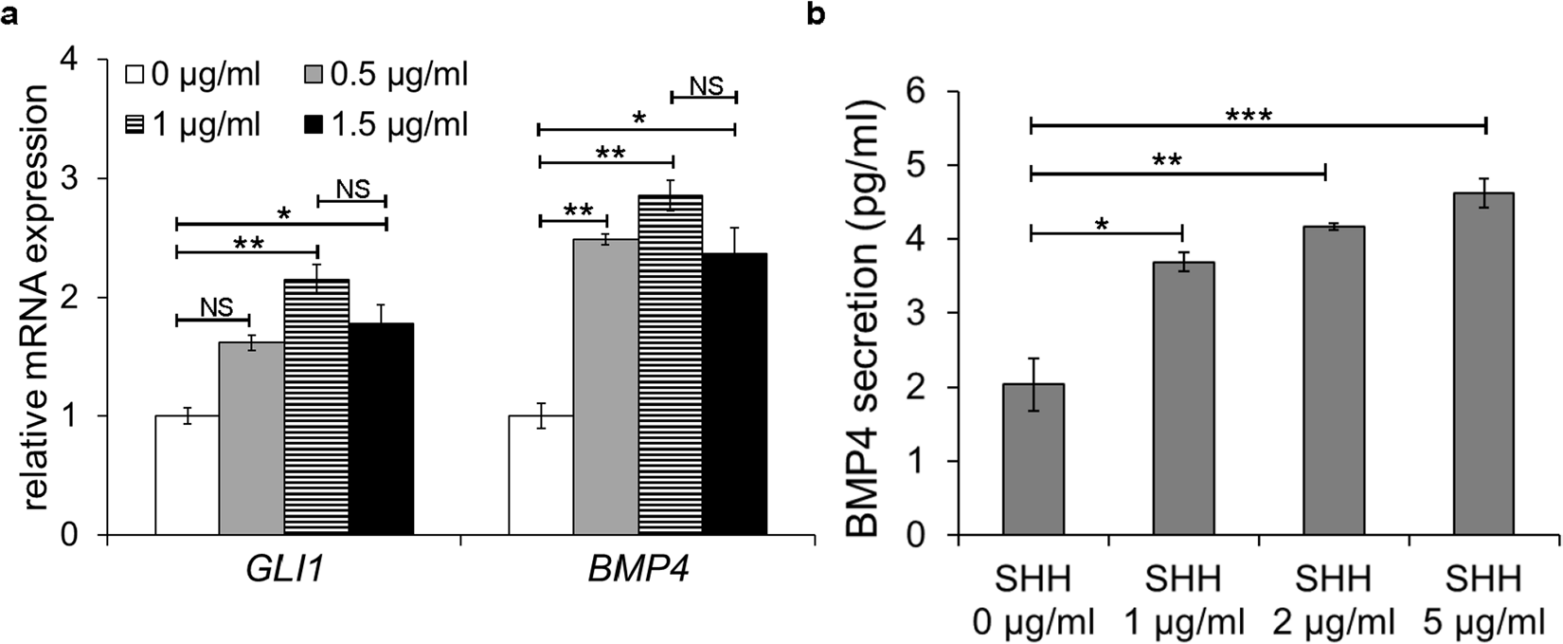
The effect of SHH treatment on HEMF *GLI1* expression and on *BMP4* expression and BMP4 protein secretion. (**a**) *GLI1* and *BMP4* expression were evaluated by qRT-PCR in HEMFs treated with increasing doses of SHH. Relative change of the target gene is shown by the 2^∆∆ (delta) CT^ method. HEMF expression of *GLI1* and *BMP4* increase in response to treatment with SHH. Results represent the means ± SEM of three individual experiments performed in HEMFs. *P ≤ 0.04; **P ≤ 0.02; NS, non-significant vs. no treatment for each gene per ANOVA for multiple comparisons (**b**) HEMF secretion of BMP4 in response to treatment with SHH was evaluated by ELISA. BMP4 secretion increases in response to treatment with SHH. Results represent the means ± SEM of three individual experiments performed in HEMFs. *P ≤ 0.02; **P ≤ 0.01; ***P ≤ 0.001 versus no treatment per ANOVA for multiple comparisons.

We were then interested in determining regulation of *BMP4* gene expression and BMP4 protein secretion in HEMFs. Given that BMP4 has been shown to be an end-target of Hedgehog (Hh) signaling in multiple other cell types^9^, we wanted to determine whether HEMFs were similarly responsive to Hh signals with BMP4 an end-target. HEMFs were treated with increasing doses of the Hh ligand SHH and expression of *GLI1*, a direct Hh target^5^ was evaluated. HEMF expression of *GLI1* mRNA increased to a peak of 2.1 fold in response to 1 µg/ml SHH and then plateaued. This finding suggested that the doses of SHH we were using were adequate to elicit a response. *BMP4* mRNA expression was then evaluated. *BMP4* mRNA increased in response to treatment with SHH and peaked and plateaued at 2.9 fold in response to a SHH dose of 1 µg/ml (Fig. 2a).

The increase in *BMP4* mRNA expression in response to SHH, prompted us to evaluate BMP4 protein secretion from HEMFs treated with increasing doses of SHH (Fig. 2b). In the absence of treatment, secretion of BMP4 from HEMFs remained near the lower detection limits of the ELISA. BMP4 secretion increased in response to increasing doses of SHH and plateaued in response to doses of SHH greater than 1 µg/ml.

### HEMFs secrete GREMLIN1

**Figure 3:**
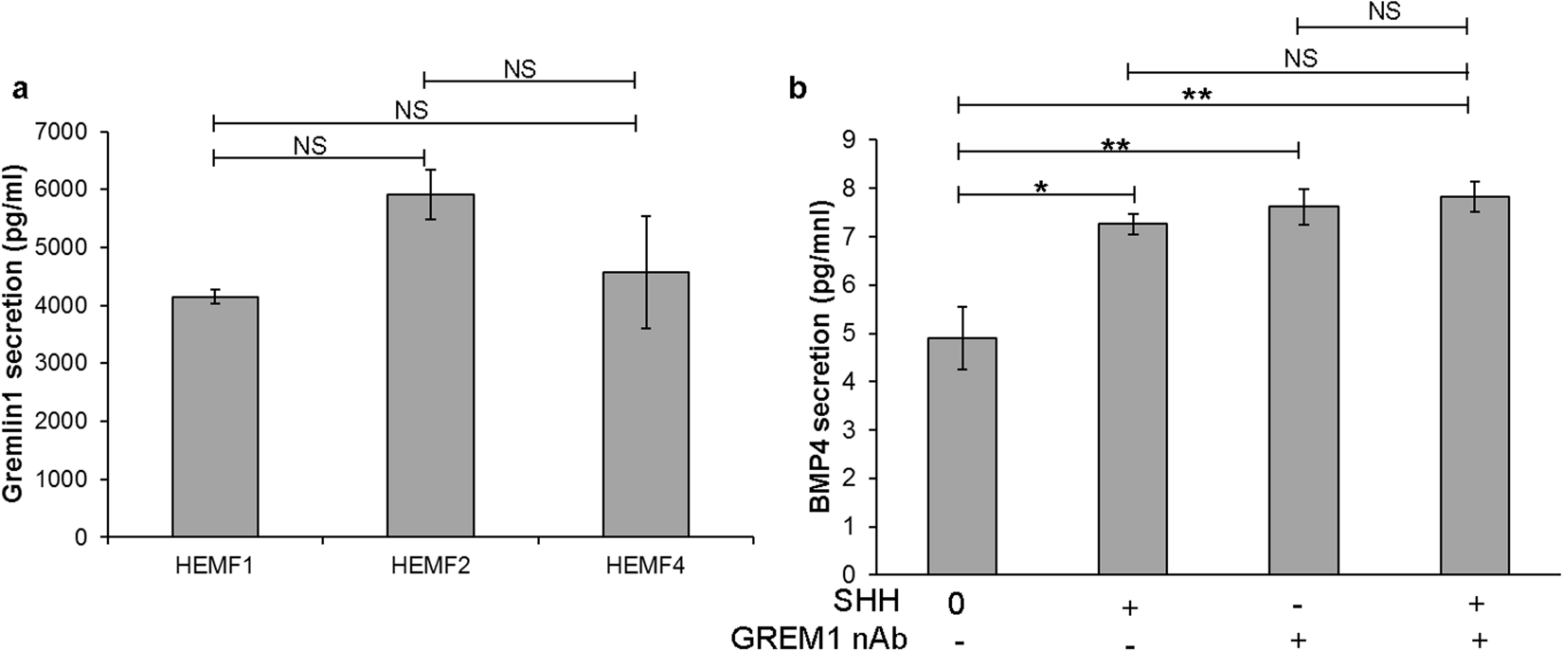
HEMF Gremlin1 secretion and BMP4 detection. (**a**) HEMFs secretion of Gremlin (GREM)1 was evaluated by ELISA performed on conditioned media of untreated HEMFs. Results represent the means ± SEM of three individual experiments performed in previously described primary cultures (HEMF 1, 2) and a GFP-hTERT transfected immortalized HEMF cell line (HEMF 5). (**b**) Detection of BMP4 in HEMF conditioned media was with ELISA after the addition of GREM1 nAb. Detection of BMP4 increased with the addition of GREM1 nAb to HEMF conditioned media, both in the presence and absence of HEMF treatment with SHH. Results represent the means ± SEM of three individual experiments performed in HEMFs. *P ≤ 0.02; **P ≤ 0.01; NS, non-significant with ANOVA for multiple comparisons.

Given our observation that GREM1 protein was expressed in cell lysates of untreated HEMFs, we were interested in whether unstimulated HEMFs secreted this BMP inhibitor and evaluated secretion of GREM1 with ELISA. High concentrations of GREM1 protein were readily detected in conditioned media collected from untreated HEMFs. ELISA results are shown for primary HEMFs 1 and 2 and HEMF 5, an immortalized cell line (Fig. 3a). HEMF GREM1 expression and secretion were then evaluated in response to SHH. GREM1 mRNA expression in HEMFs was unchanged in response to SHH. GREM1 protein continued to be detected in conditioned media collected from HEMFs treated with SHH, however the response to SHH was variable and not reproducible. Given the consistently high levels of GREM1 detected in untreated HEMF conditioned media, we were curious whether HEMF GREM1 secretion interfered with detection of stimulated BMP4 secretion. Addition of GREM1 nAb to untreated or SHH treated HEMF conditioned media similarly slightly increased detection of BMP4 (Fig. 3b).

### HEMF secreted factors increase squamous epithelium thickness

**Figure 4:**
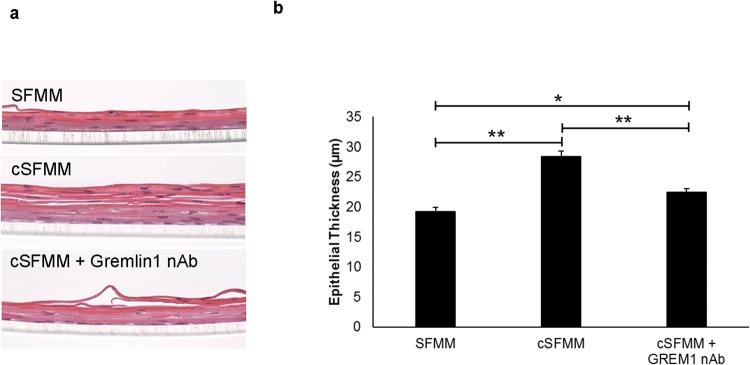
HEMF secreted factors increase epithelial cell thickness in 3D organotypic ALI culture. (**a**) H&E immunostaining of squamous epithelium harvested from 3D organotypic ALI culture shows the squamous epithelium in the presence of serum-free myofibroblast media (SFMM), conditioned serum-free myofibroblast media (cSFMM), or cSFMM plus the addition of GREM1 nAb. (**b**) Quantification demonstrates an increase in epithelial thickness 3D organotypic ALI culture with conditioned HEMF media. The increase in thickness is partially reversed with addition of GREM1 nAb. *P ≤ 0.02; **P ≤ 0.0001 with ANOVA for multiple comparisons.

We were then interested in whether conditioned HEMF media had any effects on the growth, proliferation, or differentiation of the squamous epithelium. A simplified 3D organotypic air-liquid interface (ALI) culture system that recapitulates stratified squamous epithelium was used to study the effect of HEMF secretion on the epithelium. Epithelial thickness was visibly greater in the presence of conditioned HEMF compared to epithelial cells in ALI culture with control keratinocyte or serum-free myofibroblast media (SFMM) (Fig. 4a). Quantification of epithelial thickness demonstrated an increase in epithelial thickness in the presence of conditioned HEMF media (19.2 µm vs. 28.4 µm, p < 0.0001) (Fig. 4b).

Based on our results that showed that untreated HEMFs secreted GREM1, we were curious whether GREM1 was playing a role in this morphology. Addition of GREM1 nAb to HEMF conditioned media resulted in a decrease in epithelial thickness (28.4 µm vs. 22.4 µm, p < 0.0001) with a persistently prominent eosin stained suprabasal differentiated epithelial layer and thickening of the pseudo-keratinized layer with occasional visible sloughing at the top of the epithelium, suggestive of excessive squamous differentiation (Fig. 4a,b). Addition of GREM1 nAb to control SFMM had no effect on the squamous epithelium

**Figure 5:**
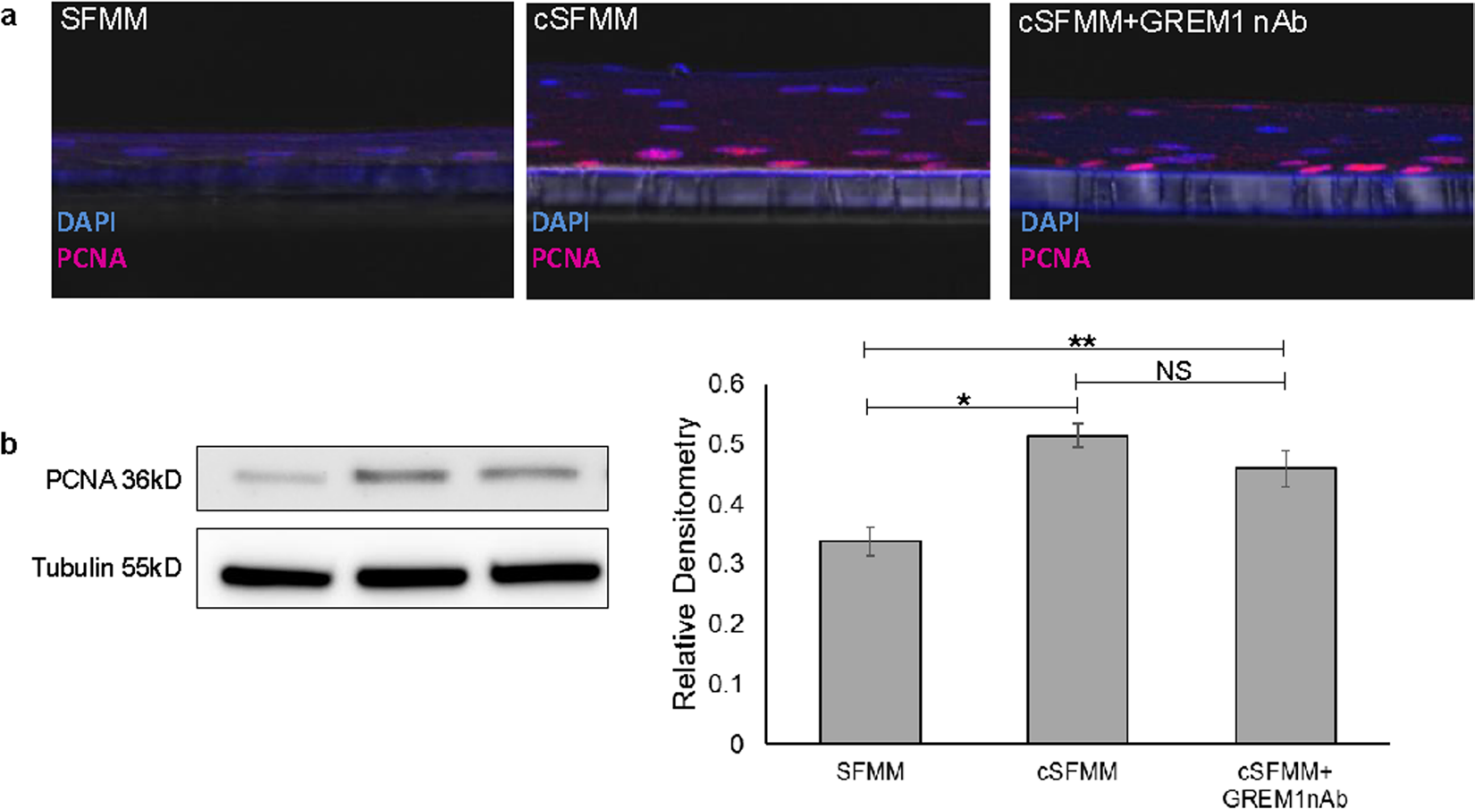
HEMF secreted factors regulate proliferation in squamous epithelial cells. (**a**) PCNA immunofluorescent staining (Cy5, purple) was performed on paraffin-embedded, formalin-fixed sections of 3D organotypic ALI culture established with control serum-free myofibroblast media (SFMM), conditioned SFMM (cSFMM), and cSFMM plus the addition of GREM1 nAb. Images were viewed with a confocal microscope. Magnification 40x. (**b**) Immunoblots for PCNA were performed on epithelium harvested from 3D organotypic ALI culture. Tubulin was used as the loading control. Representative images are shown of at least three individual 3D organotypic ALI cultures for each condition and their respective immunoblots. Samples from each experiment were derived at the same time and processed in parallel. Relative densitometry to loading control tubulin is shown. *P ≤ 0.01; **P ≤ 0.05; NS, non-significant with ANOVA for multiple comparisons.

To investigate whether an increase in basal cell proliferation was responsible for the increase in epithelial thickness, we initially performed Ki67 immunostaining. Ki67 immunostaining was sparse under all conditions precluding further reliable quantification (Supplementary Fig. 1). We therefore, evaluated expression of another proliferation marker PCNA. Immunostaining and immunoblot for PCNA demonstrated an increase in epithelial proliferation with conditioned HEMF media. PCNA expression remained increased in the presence of GREM1 nAb (Fig. 5a,b).

**Figure 6:**
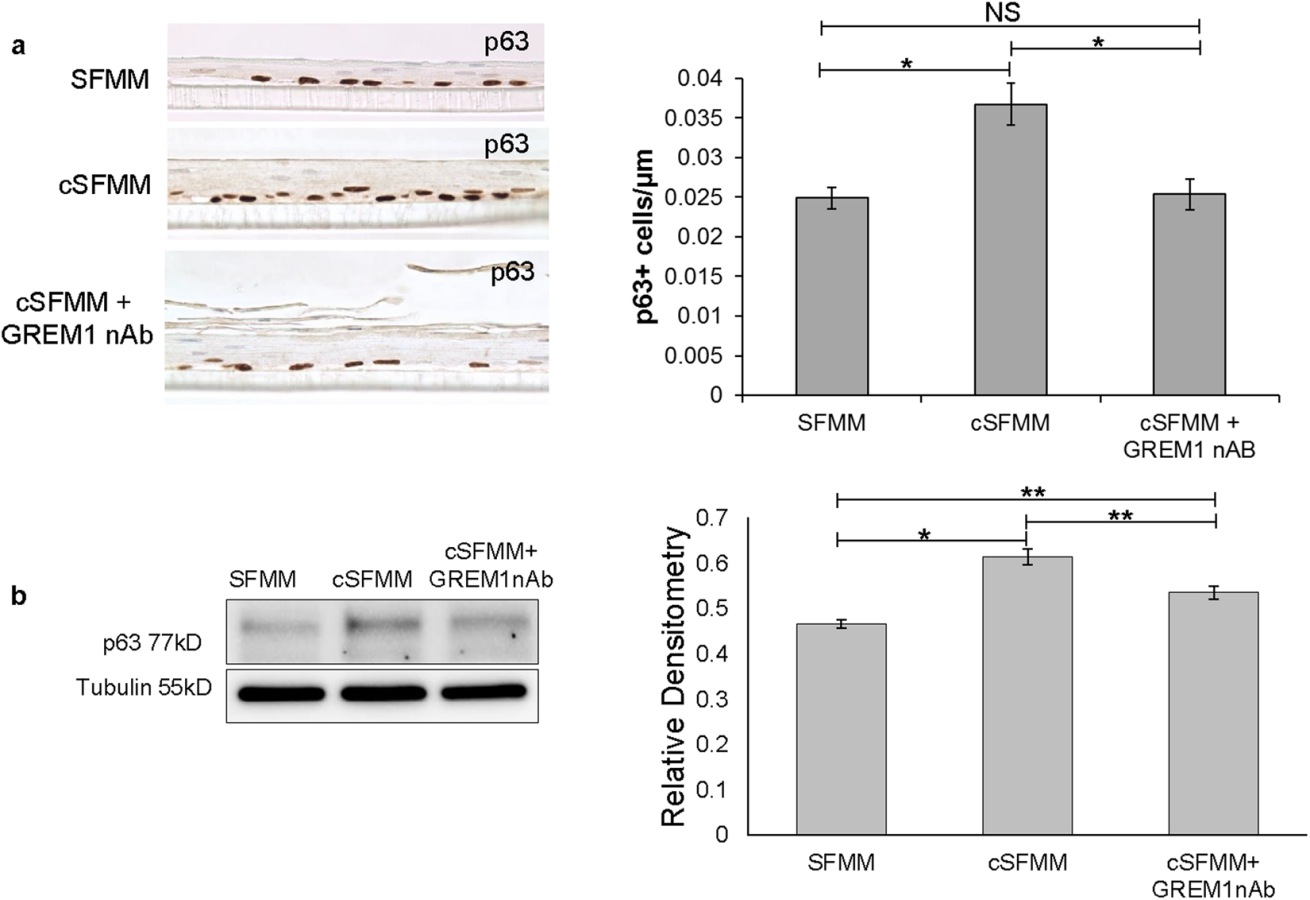
HEMF secreted factors increase epithelial basal cell p63 expression in 3D organotypic ALI culture. (**a**) Representative immunostaining for p63 performed in epithelium harvested from organotypic cultures is shown (n = 3 for each condition). Basal p63 expression in squamous epithelium is increased in the presence of conditioned HEMF media (cSFMM) compared to control (SFMM). Immunostaining for p63 is decreased in the presence of GREM1 nAb. Quantification is shown. (**b**) Immunoblot for p63 was performed on protein isolated from squamous epithelial cells harvested from 3D organotypic ALI culture. Tubulin was used as the loading control. Image shown is representative of at least three individual 3D organotypic ALI cultures for each condition and their respective immunoblots. Samples from each experiment were derived at the same time and processed in parallel. Epithelial p63 expression increases in the presence of cSFMM and partially decreases with the addition of GREM1 nAb. *P ≤ 0.01; **P ≤ 0.02; ***P < 0.05 with ANOVA for multiple comparisons.

To further characterize the population of cells that accounted for the increase in thickness and proliferation of the squamous epithelium, we performed immunostaining for the basal cell marker p63^12^. An increase in p63 immunostaining was visible in the basal layer of the squamous epithelium in the presence of conditioned vs. control HEMF media (Fig. 6a). Immunoblot confirmed the increase in p63 expression in the presence of conditioned HEMF media (Fig. 6b). In the presence of GREM1 nAb, p63 expression decreased compared to conditioned HEMF media, although its expression remained greater than that in control conditions.

**Figure 7:**
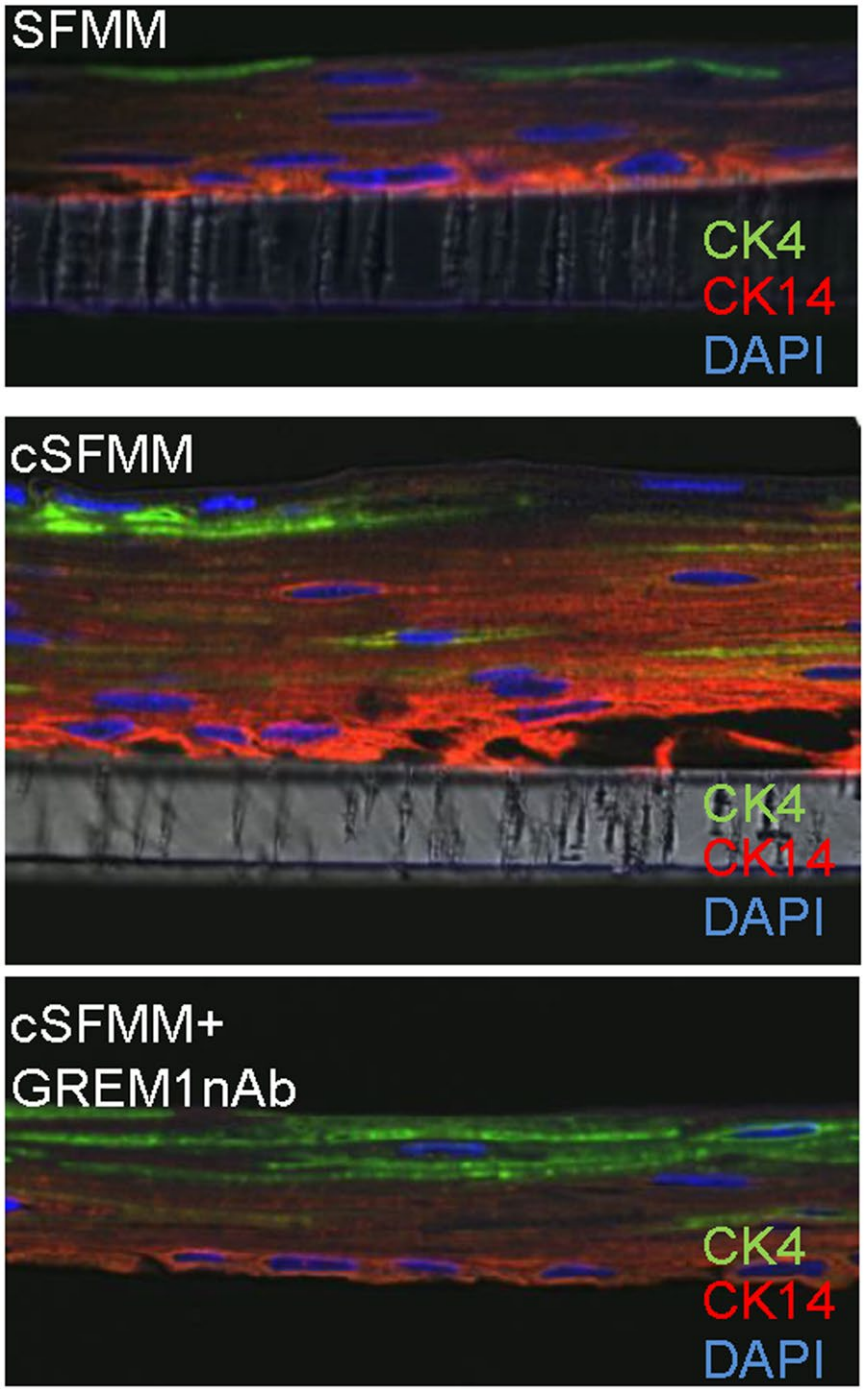
Differentiation of epithelial cells in 3D organotypic ALI culture in the presence of conditioned HEMF media and GREM1 nAb. Representative confocal images of immunostaining performed for basal cell marker CK14 (rhodamine; red) and suprabasal marker CK4 (Cy2; green) in squamous epithelium cultured with control serum-free myofibroblast media (SFMM), conditioned SFMM (cSFMM) and cSFMM plus GREM1 nAb (n = 3 each condition) are shown. Magnification 63X. Expression of basal marker CK14 is more prominent with cSFMM compared to control. Addition of GREM1 nAb decreases intensity of immunostaining for basal marker CK14 and increases intensity of suprabasal marker CK4.

To further evaluate the effect of HEMF conditioned media on expression of basal cell markers and on squamous differentiation we performed immunostaining for basal cell marker CK14 and differentiated suprabasal cells with CK 4, respectively^13^ (Fig. 7). We observed that the compared to control conditions, squamous epithelium in the presence of conditioned HEMF media had more prominent CK14 expression consistent with expansion of the basal layer. This finding was consistent with the observed increase in basal cell marker p63 expression with conditioned HEMF media. In the presence of GREM1 nAb, immunostaining for the basal layer was less intense compared to conditioned media with more prominent CK4 staining of the differentiated suprabasal layer.

### HEMF secretion modulates esophageal epithelial BMP signals

**Figure 8:**
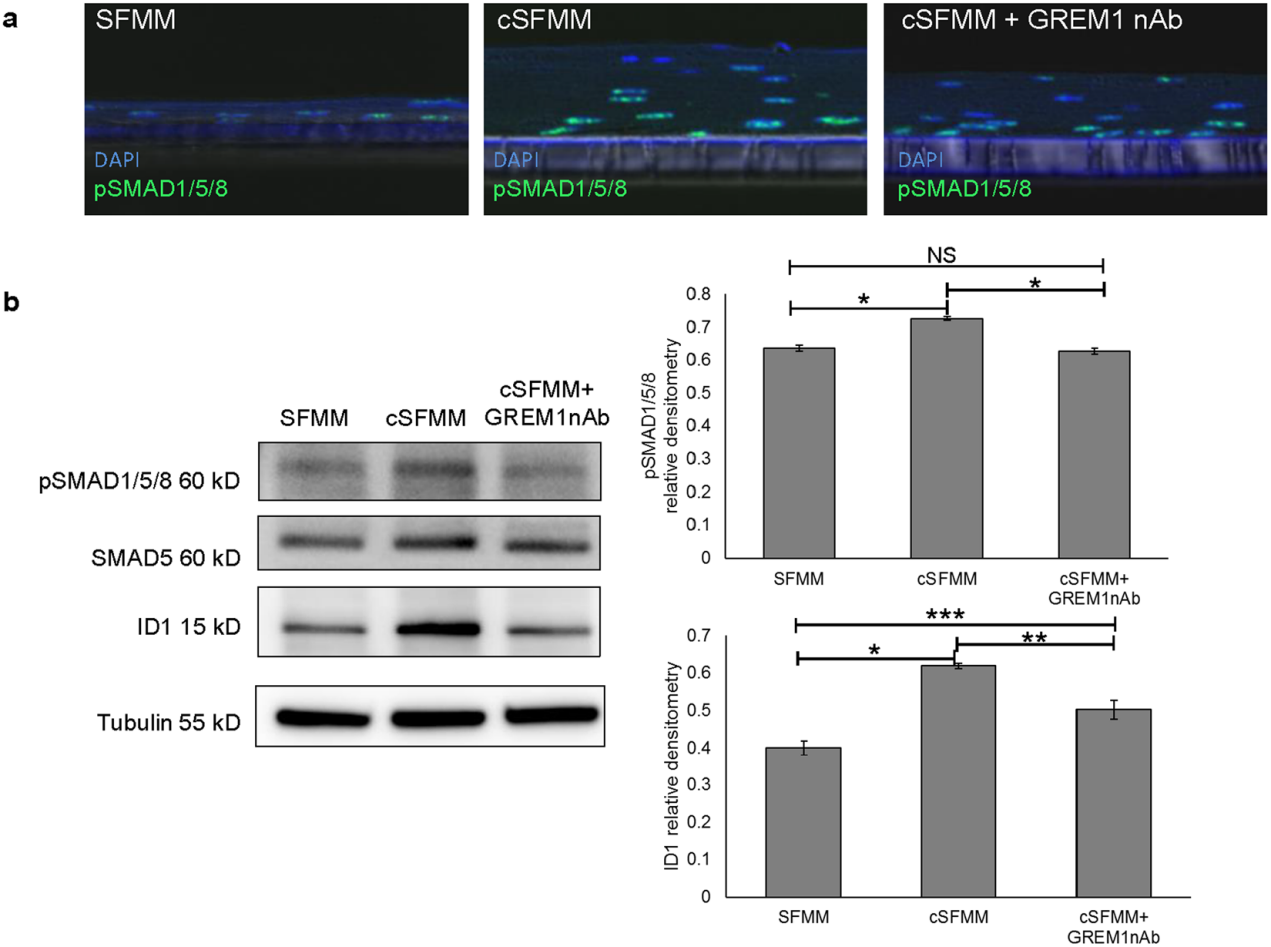
HEMF secreted factors modulate BMP signaling in squamous epithelial cells. (**a**) pSMAD1/5/8 immunofluorescent staining (Cy2, green) was performed on paraffin-embedded, formalin-fixed sections of 3D organotypic ALI culture established with control serum-free myofibroblast media (SFMM), conditioned SFMM (cSFMM), and cSFMM plus the addition of GREM1 nAb. Representative images of three independent experiments is shown. Images were viewed with a confocal microscope. Magnification 40x. (**b**) Immunoblots for pSMAD1/5/8 and ID1 were performed on epithelium harvested from 3D organotypic ALI culture. Epithelial pSMAD1/5/8 and ID1 increase in the presence of cSFMM. Addition of GREM1 nAb inhibits the increase in pSMAD1/5/8 and ID1. Tubulin was used as the loading control. Representative immunoblots are shown of three individual experiments. Samples from each experiment were derived at the same time and processed in parallel. Relative densitometry to loading control tubulin is shown. *P ≤ 0.001; **P ≤ 0.02; **P ≤ 0.05; NS, non-significant with ANOVA for multiple comparisons.

Given that GREM1 is a BMP inhibitor, we were curious whether phenotypic changes in the epithelium were a consequence of changes in BMP signaling. We therefore evaluated down-stream BMP targets in the squamous epithelium (Fig. 8a). Immunofluorescent staining for pSMAD1/5/8 showed weak expression of nuclear pSMAD1/5/8 in scattered cells of the squamous epithelium under control conditions. In the presence of conditioned HEMF media, pSMAD1/5/8 expression was intensely expressed in the nuclei of epithelial cells with occasional absent staining in the suprabasal layer. Immunofluorescent staining for pSMAD1/5/8 appeared to be weaker with the addition of GREM1 nAb to HEMF conditioned media. To better quantify pSMAD1/5/8 expression we performed immunoblot. Baseline expression of pSMAD1/5/8 increased in the presence of conditioned HEMF media. Inhibition of GREM1 decreased pSMAD1/5/8 expression similar to that in controls (Fig. 8b)

We then looked at the transcriptional target of pSMAD1/5/8, ID1^14^. Immunoblot demonstrates ID1 expression in the squamous epithelium established with control serum-free myofibroblast media. In the presence of conditioned HEMF media, baseline expression of ID1 also significantly increases. Expression of epithelial ID1 partially decreased with inhibition of GREM1 in HEMF conditioned media (Fig. 8b).

To begin to determine whether other HEMF secreted factors that could affect the BMP pathway were responsible for the observed changes in the squamous epithelium, we performed a screen for BMPs in HEMF conditioned media using a BMP array. Absence of BMP4 in conditioned media of untreated HEMFs was confirmed. BMPs 2, 5, 6, 7, 8, 9 and 11 were also not detected in conditioned media of untreated HEMF. We also performed 3D organotypic air-liquid interface (ALI) culture with the addition of BMP4 nAb to HEMF conditioned media. As expected, addition of BMP4 nAb to HEMF conditioned media did not result in morphologic changes to the squamous epithelium (Supplementary Fig. 2).

## Conclusions

In this study, we investigated the contribution of previously characterized sub-epithelial mucosal HEMFs to the BMP pathway in the human esophagus, focusing on HEMF secretion of BMP4 and its inhibitor GREM1. In addition, we adapted HEMF secreted factors into a 3D organotypic model, which recapitulates stratified squamous epithelium, thereby providing a platform for understanding HEMF paracrine interactions with the esophageal epithelium.

In humans, esophageal biopsies show an increase in BMP4 expression in esophagitis^2^ and Barrett’s esophagus^2,3^ either by immunohistochemistry or mRNA, with no detection in normal human esophagus^15^. Our findings are consistent with these previous reports. Despite *BMP4* mRNA expression in multiple cultures of HEMFs isolated from normal human esophagus we were unable to detect BMP4 protein expression or secretion from unstimulated HEMFs. Unstimulated HEMFs do not appear to be a source of BMP4. A BMP screen also did not show expression of other BMPs in unstimulated HEMF conditioned media.

We have shown, however, that BMP4 expression and secretion increase from HEMFs in response to Hh ligand SHH in a dose response manner. *BMP4* mRNA expression plateaus above doses of 1 µg/ml, possibly due to a saturation effect. Upregulation of SHH expression is induced in epithelial cells in response to acid or bile exposure^3^. In disease states such as Barrett’s esophagus, normally absent epithelial Hh ligands are reactivated in metaplastic tissue along with BMP4 expression by mesenchymal cells^16^. We found that normal HEMFs respond to SHH with an increase in *GLI1* and *BMP4*, known downstream targets of Hh signaling, demonstrating that HEMFs have the potential to be active participants in epithelial-stromal paracrine signaling in esophageal acid disorders.

Secreted active BMP4 can be inhibited at the extracellular level by interacting with secreted BMP4 antagonists such as GREM1^6^. High levels of Gremlin expression have been reported in neurons, alveolar epithelial cells, and goblet cells^17^ and upregulation of GREM1 has been reported in fibrosis of the kidney, lung, heart, liver, and pancreas^18^. Immunohistochemistry performed on tissue microarrays has shown negative or weak GREM1 expression in the human esophagus^19^. However, expression of GREM1 in the esophageal stroma at the cellular level has not been previously reported in the normal human esophagus. We have demonstrated abundant *GREM1* mRNA expression, GREM1 protein levels and secretion in HEMFs from the normal human esophagus.

Despite adequate stimulation by SHH, the quantity of BMP4 secretion as detected by ELISA in our hands remained low, suggesting possible interference from secreted GREM1. Our finding that detection of BMP4 was increased in the presence of GREM1 nAb is consistent with this possibility.

The molecular mechanisms by which GREM1 interferes with BMP activity were not fully addressed in the current study. However it is worthwhile to note that in addition to directly binding BMP4 to interfere with its interaction with the BMP receptor, Gremlin can interact with BMP4 precursor protein intracellularly and thereby inhibit the formation and secretion of mature and active BMP ligand in a tissue-specific manner^17^. Whether this mechanism is also present in HEMFs is not known. Interestingly, GREM1 may also participate in signaling pathways with endothelial and immune cells independent of the BMP pathway via interactions with VEGF receptors on endothelial cells or to Slit proteins in monocytes^8^. Furthermore, HEMFs may express additional secreted BMP antagonists that were not investigated in the current study.

We investigated the functional consequences of unstimulated HEMF secretion in an organotypic model in which upper chamber epithelial cells are co-cultured with conditioned myofibroblast media in the lower chamber to determine the effect of HEMF secreted factors on the epithelium. Epithelial cells are exposed to HEMF conditioned media at the time of initiation of the air-liquid interface (i.e. *after* epithelial cells have been seeded atop the transwell membrane and have already begun to proliferate). We observed that secreted factors in HEMF conditioned media increase epithelial thickness via an increase in epithelial proliferation and an increase in basal layer expansion, as reflected by the increase in basal cell markers p63 and CK14. We also observed an increase in epithelial pSMAD1/5/8 and ID1 in the presence of HEMF conditioned media, suggesting that secreted factors activate BMP signaling in epithelial cells. It is not clear from our results, however, that this increase is responsible for the observed increase in epithelial thickness and proliferation. For instance, BMP activation in the epithelium has recently been shown to promotes differentiation and not proliferation of basal progenitor cells in the esophagus^4^, suggesting the possibility that in our model, the increase in epithelial BMP signaling is a secondary response to activation of as yet uninvestigated pathways involved in the regulation of epithelial proliferation and differentiation (e.g. Wnt, Notch).

Given abundant GREM1 secretion from cultures of HEMFs we hypothesized that GREM1 was mediating some of these observed effects. GREM1 inhibition only partially decreased epithelial thickness and expression of p63 with a trend towards a decrease in epithelial proliferation. GREM1 inhibition also resulted in a squamous epithelium that had a more prominent differentiated suprabasal layer and less prominent basal layer. Furthermore, the increase in epithelial BMP signaling despite abundant HEMF GREM1 secretion and the incongruous decrease in BMP activation with GREM1 inhibition demonstrate the potential role of additional uninvestigated factors in HEMF conditioned media. Overall, our findings suggest that other as yet unidentified factors in conditioned HEMF media contribute to the observed phenotype. In addition, our work suggests that the effect of HEMF GREM1 secretion in our model may not be primarily via its role as a BMP inhibitor and suggests the possibility of a direct effect of GREM1 on the squamous epithelium. Others, for instance, have previously reported on the functions of GREM1 as a regulator of survival and proliferation independent of its effects on the BMP pathway^20^.

Our work has several limitations including the use of HEMF cell cultures and immortalized HEMF cell lines and limitations of the 3D organotypic model. Although HEMFs were derived from normal human esophagus, and in theory more reflective of human physiology than mouse cells, they are cells and may not fully recapitulate *in vivo* physiology. Furthermore, there may be underappreciated differences in secretion from primary cultures of HEMFs vs. from a cell line, although this cell line has been previously shown to behave similar to primary HEMFs^11^. While absolute expression values are expectedly different amongst primary cultures themselves and between primary cultures and cell line, the patterns are similar. We looked at the functional consequences of HEMF secretion in a modified 3D organotypic model using HEMF conditioned media. This model utilizes conditioned media from a well characterized HEMF cell line and does incorporate HEMFs into the collagen matrix of the traditional 3D OTC model. While stratified squamous epithelium is recapitulated in both models, the physical contribution of HEMFs working in concert with HEMF secreted factors is lacking. In addition, the squamous epithelium was only at the time of harvest on day 14 of culture. Off-target effects of the neutralizing antibody used may also be possible, although no difference in epithelial morphology was observed with addition of GREM1 to control serum-free myofibroblast media. These limitations along with investigation of the effect of stimulated HEMF secretion on the epithelium should be addressed in ongoing studies of paracrine interactions between HEMFs and the squamous epithelium using genetically modified HEMFs in both traditional and modified 3D OTC.

**Figure 9:**
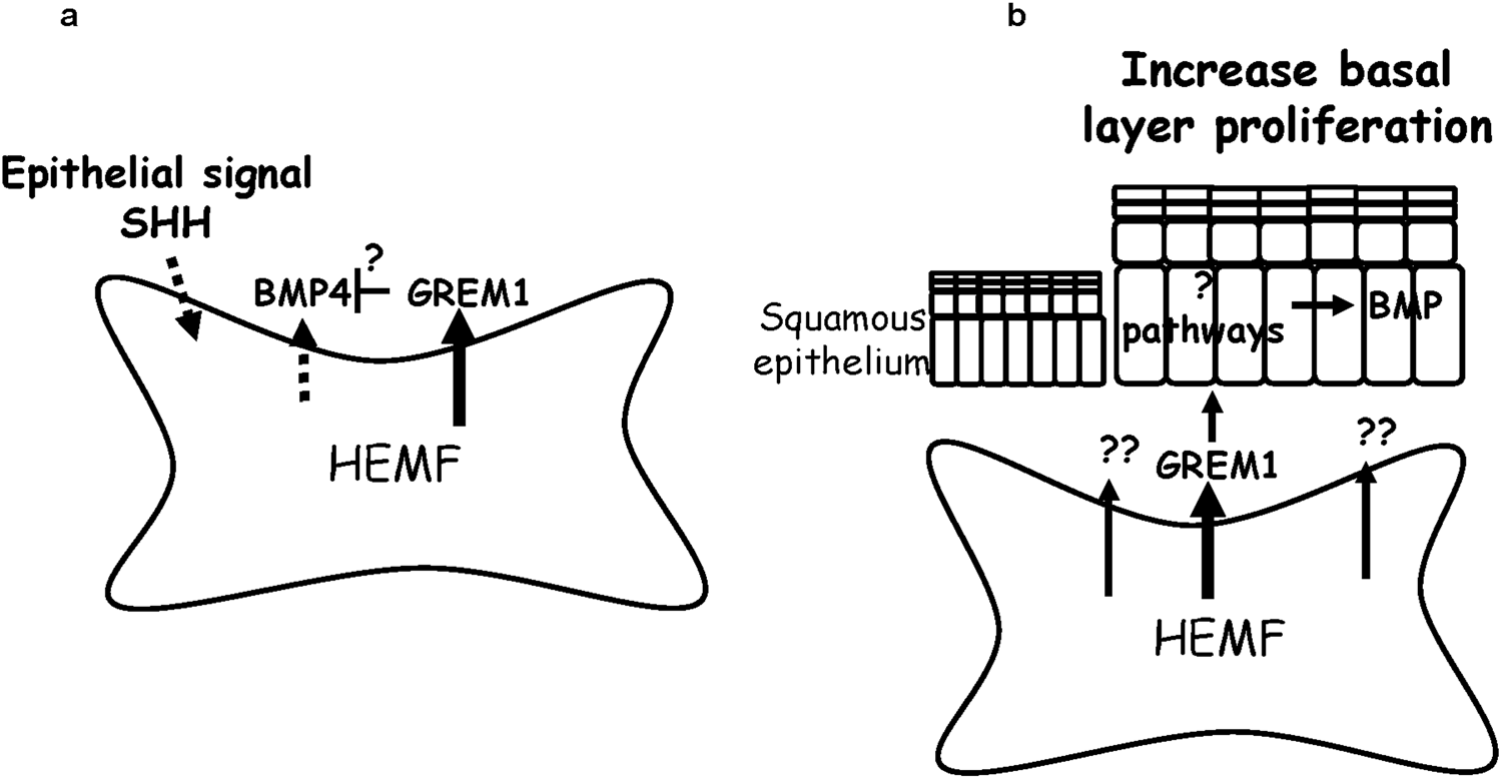
Schematic of proposed interactions between (a) SHH signaling and HEMF secretion of BMP4 and GREM1 and between (b) HEMF secreted factors, epithelial phenotypes and BMP signaling. (**a**) Untreated HEMFs constitutively secrete GREM1 and secrete BMP4 in response to SHH. GREM1 secretion may interfere with detection of BMP4 in stimulated HEMFs. (**b**) Unstimulated HEMF secreted factors increase epithelial cell thickness and basal cell proliferation in part via GREM1 secretion with contribution of additional unidentified factors. The effects of GREM1 in this model may be a direct effect on the squamous epithelium. Activated BMP signaling in the squamous epithelium may be a secondary consequence of unidentified pathway. HEMF, human esophageal myofibroblast.

Investigation of human esophageal pathobiology has historically been limited to the squamous epithelium with little consideration of the microenvironment. Furthermore, profound differences between mouse and human esophagus may limit generalizability of signaling pathways detailed in the mouse esophagus and necessitate evaluation in human cells and tissue. Despite its limitations, our work is one of the first to look at BMP signaling *in the HEMFs* of the human esophagus and paracrine interactions between HEMFs and the squamous epithelium. We have demonstrated for the first time that HEMFs are a potential source of BMP4 in the setting of stimulation and that HEMF basal secretion of GREM1 secretion reduces detection of BMP4 (Fig. 9a). Our work shows that HEMF secreted factors regulate epithelial thickness in a 3D organotypic model at least in part via GREM1 secretion through as yet undetermined mechanisms, including the possibility of a direct effect on the epithelium (Fig. 9b). Our findings support additional investigation of the effects HEMF secretion on the squamous epithelium in normal and diseased esophagus.

## Methods

### Cell culture

Previously characterized primary HEMF cultures from normal human esophagi (HEMFs 1–3)^10^, a freshly generated HEMF culture (HEMF 4) from de-identified normal human esophageal specimen from donor without a known history of esophageal disorders, and an immortalized HEMF cell line (HEMF 5)^11^ with phenotypic, genotypic and functional similarity to primary cultures were cultured in myofibroblast media (DMEM with 10% FBS supplemented with 10 mg/ml insulin; and 10 µg/ml gentamicin, 2 ng/ml EGF, and 10 µg/ml transferrin) as previously described^10^ and incubated in 37 °C with 5% CO2. A well characterized human esophageal epithelial cell line EPC2 (telomerase reverse transcriptase-immortalized, gift from Dr. Anil Rustgi, University of Pennsylvania, Philadelphia, PA) cultured in keratinocyte serum-free media with 0.09 mM CaCl_2_ and supplemented with BPE and penicillin-streptomycin as previously described^21^. This protocol was approved by the Institutional Review Board of Keck School of Medicine of University of Southern California and deemed coded specimens/data. All methods were performed in accordance with relevant guidelines and regulations.

### Treatment of HEMFs with SHH and ELISA

For treatment studies, HEMFs were grown in serum-free myofibroblast media (SFMM). Preliminary studies showed a similar response pattern to SHH treatment between HEMF primary cultures and immortalized cell line HEMF 5. Therefore, HEMF 5 was used for signaling studies and treated with SHH (0.5–5 µg/ml) and Gremlin (GREM) 1 neutralizing antibody (nAb) for 24 h. Pilot studies performed to determine the optimal concentration of Grem1 nAb showed that 3 µg/ml inhibited detection of Grem1 in HEMF conditioned media. This concentration was therefore used in subsequent studies. Conditioned media from untreated HEMFs was collected for evaluation of BMPs with the RayBio C-Series human BMP Related Array 1 according to the manufacturer’s instructions. Conditioned media from treated HEMFs was collected for evaluation of BMP4 (R&D Duoset, DY314) and Grem1 (Lifeome, E92128Hu-1) using enzyme-linked immunosorbent assay (ELISA) according to the manufacturer’s instructions. Protein levels in each sample were determined based on a standard curve generated by recombinant proteins provided with the ELISA kits. Results were expressed as the means ± SEM in pg/ml.

### RNA isolation and rt-PCR

RNA was harvested from HEMFs using GeneElute Mammalian Total RNA miniprep Kit (Sigma-Aldrich, St. Louis, MO) per manufacturer’s recommendations and further cleaned with DNA-free (Ambion, Austin, TX). cDNA was synthesized and mRNA expression of the following genes was evaluated: BMP4 (5′ CTGGTCTTGAGTATCCTGAGCG 3′; 3′CTGGTCTTGAGTATCCTGAGCG 5′), GREM1 (5′ TCATCAACCGCTTCTGTTACGGC 3′; 3′ CAGAAGGAGCAGGACTGAAAGG 5′), GREM2 (5′ TTTCCCTGTCCTTGTTCCTG 3′; 3′ TGCACCAGTCACTCTTGAGG 5′)and Gli1 (5′ GCCGTGCTAAAGCTCCAGTGA 3′; 3′ CTGCCCTATGTGAAGCCCTATTTG 5′). Human 18 s (5′ CCA TGA AGA GGT GAG CGG GGA TTG; 3′ ATT AAG TCC CTG CCC TTT GTA CAC 5′) was used as an endogenous control to normalize the samples using the ∆Ct and ∆∆Ct methods of relative quantitation, where Ct is the critical threshold cycle. Target gene expression was normalized by Ct of the endogenous control (18 s rRNA) from that of the target gene (ΔCT). For comparisons, relative change of the target gene equals 2^ΔΔCT^, where ΔΔ CT = ΔCT of the tested sample - ΔCT of the control sample. Experiments included no template and no reverse transcriptase controls for each gene.

### 3D organotypic Air-liquid interface culture

A 3D organotypic air-liquid interface (ALI) culture protocol was performed as previously described^22^. Briefly, immortalized esophageal epithelial cells (EPC2-hTERT) were seeded onto semi-permeable membranes (0.4 μm, Corning, Corning NY) and grown to confluence in the presence of low-calcium media (keratinocyte serum free media, 0.09 mM calcium). Epithelial differentiation was induced by the addition of extracellular calcium (1.8 mM final concentration) for the next five days (day 3 to 8). Stratification was induced by removing the media from the upper chamber and exposing the cells to the ALI for a period of 6 days (day 8 to 14). At the start of the ALI exposure (day 8), different treatment regimens were added to the lower chamber and continued until the day of harvest (day 14). Epithelial cells had a similar morphology with keratinocyte serum free media and serum-free myofibroblast media (SFMM) in the lower chamber. SFMM therefore served as the control medium and comparator in subsequent studies. Treatment regimens included HEMF conditioned SFMM (cSFMM) and HEMF conditioned media containing Gremlin1 nAb (cSFMM + GREM1 nAb). Conditioned media was prepared by growing 8 × 10^5^ immortalized HEMFs in serum-free myofibroblast medium in a 75 cm^2^ flask with and without the addition of GREM1 nAb and collection of media after 24 hours. Lower chamber media was replaced with fresh conditioned media every 3 days. Cultures were fixed with 10% neutralized buffered formaldehyde in the plate at 4 °C for 1 hour and submitted for histology.

### Protein isolation and immunoblot

The squamous epithelium in 3D ALI culture was harvested by stripping the epithelial layer off the membrane with forceps. Cells were collected into RIPA lysis buffer and sonicated. Protein was quantified by the Bradford assay; 20 µg of whole cell lysates were subjected to 4–20% SDS-PAGE gel and transferred to PVDF membrane. The membrane was blocked with 5% milk or BSA and incubated overnight with rabbit anti-p63 (0.33 µg/ml, Genetex, #GTX102425), mouse anti-PCNA (1:400, BD Transduction, cat#610664), rabbit anti-pSMAD1/5/8 monoclonal (1:500, Cell Signaling, cat# 9516), rabbit anti-SMAD5 monoclonal (1:1000, Cell Signaling, cat# 12534 S), or rabbit anti-ID1 polyclonal antibody (0.5 µg/ml, Santa Cruz #sc-488). Membranes were washed with TBST and incubated with donkey anti-mouse-HRP (1:5000, Santa Cruz, cat#sc-2314) or donkey anti-rabbit IgG-HRP (1:5000, Santa Cruz, cat#sc-2317) for 1 hour at room temperature. Immunoreactive bands were detected by SuperSignal West Pico PLUS chemiluminescent substrate (Thermo Scientific, cat#34577). Membranes were stripped and reprobed with mouse anti-tubulin (1:5k,Santa Cruz, sc-5286) or rabbit β-actin polyclonal (1:10k, Abcam (ab8227) antibodies as loading controls. Protein expression was quantified using NIH Image J software and the relative quantity of protein with respect to tubulin was calculated.

### Immunohistochemistry and immunofluorescence

Paraffin-embedded, formalin-fixed sections of 3D organotypic ALI cultures were used for immunohistochemistry and immunofluorescence as previously described^10^. Primary antibodies used for immunohistochemistry were mouse monoclonal anti-p63 (1:200, BioCare Medical, CM163A] and rabbit polyclonal anti-Ki-67 (1:500, Leica Biosystems Ki67P-CE). The slides were examined with a Nikon microscope. To quantify epithelial thickness, 5 µm sections stained with hematoxylin-eosin and evaluated under a microscope (Nikon) with a 40x objective. For each field of vision, epithelial thickness was measured from each end of the visible strip of tissue as well as the middle, for a total of 3 measurements. The entire length of each available strip was evaluated in this manner. p63 positive cells were counted in each field of vision (FOV) under the microscope at 40x. The length of the epithelial cell strip in each FOV was measured and the number of p63-positive cells counted. The number of p63+ cells per um of tissue was then calculated. The primary antibodies used for immunofluorescence were mouse anti-CK14 (1:600, Abcam, ab7800), rabbit anti-CK4 (1:100, Abcam, ab51599), mouse anti-PCNA (1:100, BD Transduction, cat#610664), rabbit anti-pSMAD1/5/8 (1:100, Cell Signaling Technology, cat# 9516) and followed by goat anti-rabbit CY2 (1:1000), goat anti-mouse CY5 (1:600), or rodamine goat anti-mouse (1:200) secondary antibodies (Jackson ImmunoResearch). Immunofluorescent slides were examined with a confocal microscope (Leice TCS SP8-Leica microsystems).

### Statistics/Analysis

All experiments were performed in triplicate and data presented as means ± SE. Data were analyzed using Student’s two-tailed type 2 t-test or ANOVA with a Tukey’s or Dunnett’s post hoc test, as appropriate with GraphPad Prism 6.0 (GraphPad Software, La Jolla, CA). A value of *P* < 0.05 indicated statistical significance.

Materials, data, and associated protocols are available upon request.

## Electronic supplementary material

Supplementary Figures

## References

[1] Rodriguez P (2010). BMP signaling in the development of the mouse esophagus and forestomach. Development.

[2] Milano F (2007). Bone morphogenetic protein 4 expressed in esophagitis induces a columnar phenotype in esophageal squamous cells. Gastroenterology.

[3] Wang DH (2010). Aberrant epithelial-mesenchymal Hedgehog signaling characterizes Barrett’s metaplasia. Gastroenterology.

[4] Jiang M (2015). BMP-driven NRF2 activation in esophageal basal cell differentiation and eosinophilic esophagitis. The Journal of clinical investigation.

[5] Rosekrans SL, Baan B, Muncan V, van den Brink GR (2015). Esophageal development and epithelial homeostasis. American journal of physiology. Gastrointestinal and liver physiology.

[6] Ali IH, Brazil DP (2014). Bone morphogenetic proteins and their antagonists: current and emerging clinical uses. British journal of pharmacology.

[7] Miyazono K, Miyazawa K (2002). Id: a target of BMP signaling. Science’s STKE: signal transduction knowledge environment.

[8] Brazil DP, Church RH, Surae S, Godson C, Martin F (2015). BMP signalling: agony and antagony in the family. Trends in cell biology.

[9] Roberts DJ (1995). Sonic hedgehog is an endodermal signal inducing Bmp-4 and Hox genes during induction and regionalization of the chick hindgut. Development.

[10] Gargus M (2015). Human esophageal myofibroblasts secrete proinflammatory cytokines in response to acid and Toll-like receptor 4 ligands. American journal of physiology. Gastrointestinal and liver physiology.

[11] Niu C, Chauhan U, Gargus M, Shaker A (2016). Generation and Characterization of an Immortalized Human Esophageal Myofibroblast Line. PloS one.

[12] DeWard AD, Cramer J, Lagasse E (2014). Cellular heterogeneity in the mouse esophagus implicates the presence of a nonquiescent epithelial stem cell population. Cell reports.

[13] van Dop WA (2013). Hedgehog signalling stimulates precursor cell accumulation and impairs epithelial maturation in the murine oesophagus. Gut.

[14] Yang J (2013). Id proteins are critical downstream effectors of BMP signaling in human pulmonary arterial smooth muscle cells. American journal of physiology. Lung cellular and molecular physiology.

[15] van Baal JW (2005). A comparative analysis by SAGE of gene expression profiles of Barrett’s esophagus, normal squamous esophagus, and gastric cardia. Gastroenterology.

[16] Wang DH (2014). Hedgehog signaling regulates FOXA2 in esophageal embryogenesis and Barrett’s metaplasia. The Journal of clinical investigation.

[17] Sun J (2006). BMP4 activation and secretion are negatively regulated by an intracellular gremlin-BMP4 interaction. The Journal of biological chemistry.

[18] Brazil DP (2015). Gremlin1 and chronic pancreatitis: a new clinical target and biomarker?. Journal of molecular medicine.

[19] Laurila R, Parkkila S, Isola J, Kallioniemi A, Alarmo EL (2013). The expression patterns of gremlin 1 and noggin in normal adult and tumor tissues. International journal of clinical and experimental pathology.

[20] Han EJ (2016). GREM1 Is a Key Regulator of Synoviocyte Hyperplasia and Invasiveness. The Journal of rheumatology.

[21] Kalabis J (2012). Isolation and characterization of mouse and human esophageal epithelial cells in 3D organotypic culture. Nature protocols.

[22] Kc K, Rothenberg ME, Sherrill JD (2015). *In vitro* model for studying esophageal epithelial differentiation and allergic inflammatory responses identifies keratin involvement in eosinophilic esophagitis. PloS one.

