# Supplementary material for: Human esophageal myofibroblast secretion of bone morphogenetic proteins and GREMLIN1 and paracrine regulation of squamous epithelial growth
Source: Sci Rep. 2018 Aug 17;8:12354. doi: 10.1038/s41598-018-30799-7 (PMC6098060; doi:10.1038/s41598-018-30799-7)

Human esophageal myofibroblast secretion of bone morphogenetic proteins and GREMLIN1 and paracrine regulation of squamous epithelial growth

Chunying Zhang[1], Chao Niu[1], Kevin Yang[1], Anisa Shaker[1*]

[1]Department of Internal Medicine, Division of Gastrointestinal and Liver Diseases, Keck School of Medicine of USC, Los Angeles, CA, USA

[*] Corresponding author

E-mail: ashaker@usc.edu

Supporting information

Supplementary Figure 1. Epithelial cell proliferation in squamous epithelium in 3D organotypic ALI culture with HEMF conditioned media. Ki-67 immunostaining was performed on paraffin-embedded, formalin-fixed sections of 3D organotypic ALI cultures established with serum-free myofibroblast media (SFMM), conditioned SFMM, and cSFMM with GREM1 nAb. Sparse Ki-67 immunostaining, limited to the basal cell layer, is seen under all conditions, precluding reliable quantification.

Supplementary Figure 2. H&E of squamous epithelium in 3D organotypic ALI culture with HEMF conditioned media with and without BMP4 nAb. H&E immunostaining of squamous epithelium harvested from 3D organotypic ALI culture shows the squamous epithelium in the presence of serum-free myofibroblast media (SFMM), conditioned serum-free myofibroblast media (cSFMM), or cSFMM plus the addition of BMP4 nAb. Epithelial squamous morphology and thickness are unchanged with the addition of BMP4 nAb to conditioned HEMF media.

Supplementary Figure 1. Basal cell proliferation in squamous epithelium in 3D organotypic ALI culture with HEMF conditioned media with and without GREM1 nAb. Sparse Ki-67 immunostaining is seen under all conditions,

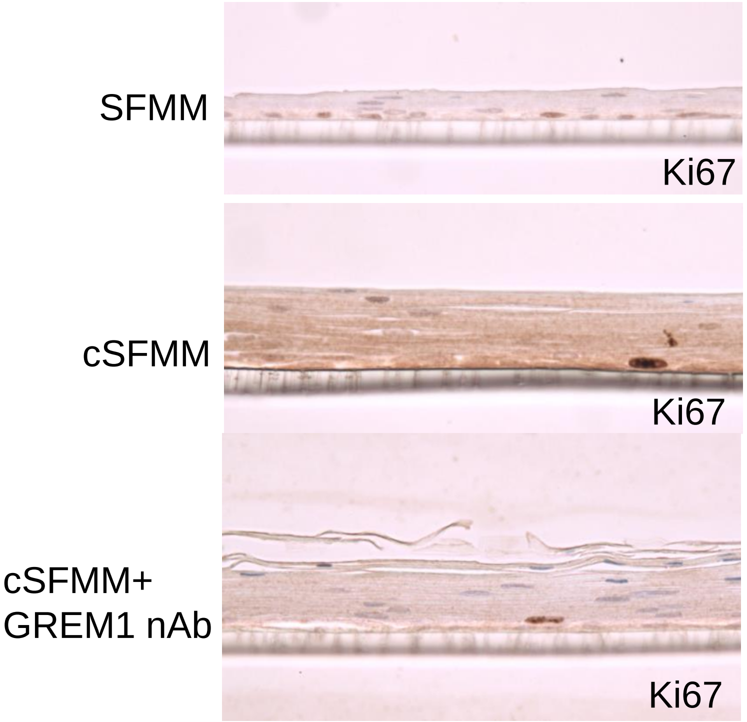

Supplementary Figure 2. H&E of squamous epithelium in 3D organotypic ALI culture with HEMF conditioned media with and without BMP4 nAb. Epithelial squamous morphology and thickness are unchanged with the addition of BMP4 nAb to conditioned HEMF media in 3D organotypic ALI culture.

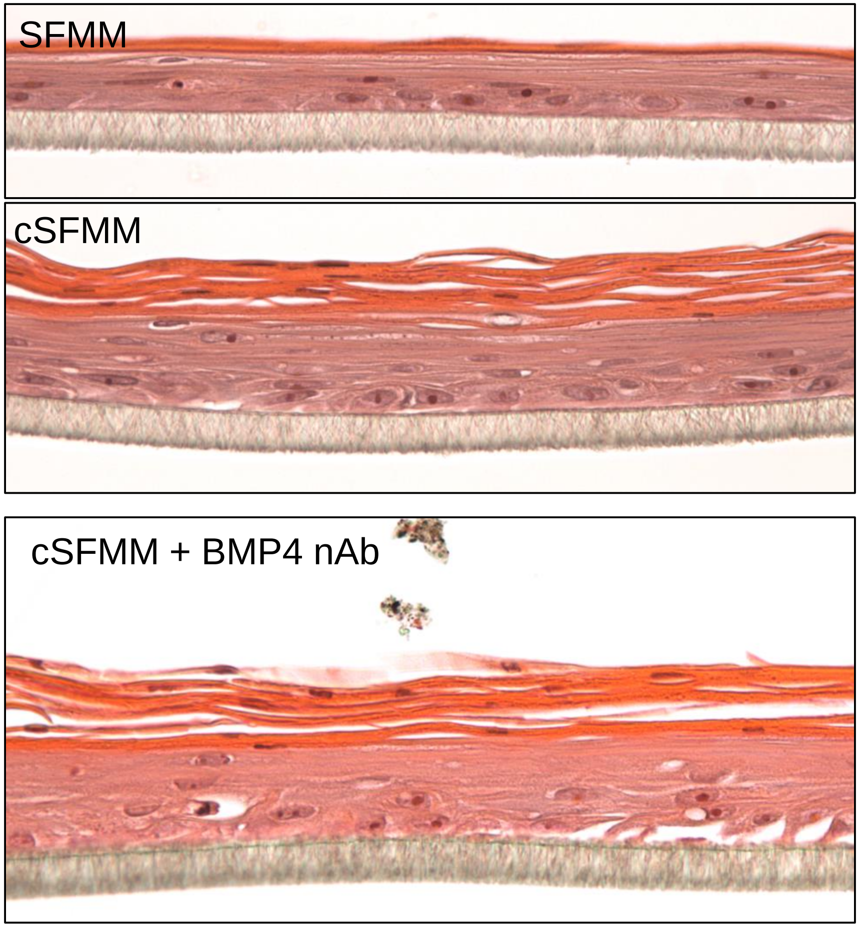

Supplement: Supplementary file 1 — Supplementary Figures [file 41598_2018_30799_MOESM1_ESM.pdf]
